# Metabolic syndrome predicts postoperative complications after gastrectomy in gastric cancer patients: Development of an individualized usable nomogram and rating model

**DOI:** 10.1002/cam4.3352

**Published:** 2020-08-17

**Authors:** Xiaodong Chen, Weiteng Zhang, Xiangwei Sun, Mingming Shi, Libin Xu, Yiqi Cai, Wenjing Chen, Chenchen Mao, Xian Shen

**Affiliations:** ^1^ Department of Gastrointestinal Surgery The Second Affiliated Hospital of Wenzhou Medical University and Yuying children's Hospital Wenzhou China; ^2^ Department of Gastrointestinal Surgery The First Affiliated Hospital Wenzhou Medical University Wenzhou China

**Keywords:** gastrectomy, metabolic syndrome, nomogram, postoperative complications

## Abstract

**Background:**

Metabolic syndrome (MetS), a public health problem, is reportedly related to an increased risk of postoperative complications after surgery. However, whether MetS have an effect on complications after gastric cancer (GC) surgery are unknown. This study aimed to investigate the effects of preoperative MetS on complications after gastrectomy.

**Methods:**

Altogether, 718 gastric cancer patients who planned to receive radical gastrectomy between June 2014 and December 2016 were enrolled, demographic and clinicopathological characteristics were analyzed. Univariate and multivariate analyses were performed to identify potential risk factors for postoperative complications. A predictive model for postoperative complications was constructed in the form of a nomogram, and its clinical usefulness was assessed.

**Results:**

Of the 628 patients ultimately included in the study (mean age 62.92 years, 450 men and 178 women), 84 were diagnosed with MetS preoperatively. Severe postoperative complications (Clavien‐Dindo grade ≥II) were significantly more common in patients with MetS (41.7% versus 23.7%, *P* < .001). Predictors of postoperative complications included MetS (odds ratio [OR] = 1.800, *P* = .023), age (OR = 1.418, *P* = .050), Charlson score (OR = 1.787, *P* = .004 for 1‐2 points) and anastomosis type (OR = 1.746, *P* = .007 for Billroth II reconstruction). The high‐risk rating had a high AUC (ROC I = 0.503, ROC Ib = 0.544, ROC IIa = 0.601, ROC IIb = 0.612, ROC IIc = 0.638, ROC III = 0.735), indicating that the risk‐rating model has good discriminative capacity and clinical usefulness.

**Conclusions:**

MetS was an independent risk factor for complications after gastrectomy. The nomogram and rating model incorporating MetS, Billroth II anastomosis, age, and Charlson score was useful for individualized prediction of postoperative complications.

## Graphical abstract

MetS was an independent risk factor for postoperative complications after gastrectomy. We developed an useful nomogram and rating model incorporating MetS, Billroth II anastomosis, age, and Charlson score.

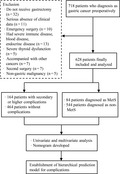

## 1 INTRODUCTION

Metabolic syndrome (MetS) is characterized by a series of metabolic disturbances including obesity, hyperglycemia, dyslipidemia, and high blood pressure.^1^, ^2^, ^3^ Recently, studies have indicated the carcinogenic function of MetS in variety cancer including liver,^4^ pancreatic,^5^ colorectal,^6^ and gastric^7^ cancers, and associations between MetS and poor prognoses have been reported in patients with these types of cancers.^2^, ^8^ A number of studies have yielded discordant results however,^9^, ^10^ thus, it remains controversial whether preoperative MetS is related to postoperative complications.

Worldwide, the incidence and lethality of the gastric cancer (GC) has increased during the past decades, GC has been the 4th common cancer and the 3rd cause of cancer‐related‐mortality.^11^, ^12^ To date surgical resection remains the preferred therapeutic approach for gastric cancer. Postoperative complications after gastrectomy affect life quality, delay subsequent therapies, and even overall survival, and despite the development of minimally invasive surgery and the enhancement of postoperative recovery program, their reported prevalence still ranges from 10% to 46%.^13^, ^14^, ^15^ Risk assessment preoperatively is therefore significant, to identify postoperative complication prone patients.

Considering that the aforementioned previous ambiguous results are probably because of retrospective research designs as well as limited patient cohorts, we conducted the present prospective multicenter study incorporating a large sample size with the aim of better evaluating the potential effects of preoperative MetS on postoperative complications after gastrectomy.

## 2 MATERIALS AND METHODS

### 2.1 Patient

**Figure 1:**
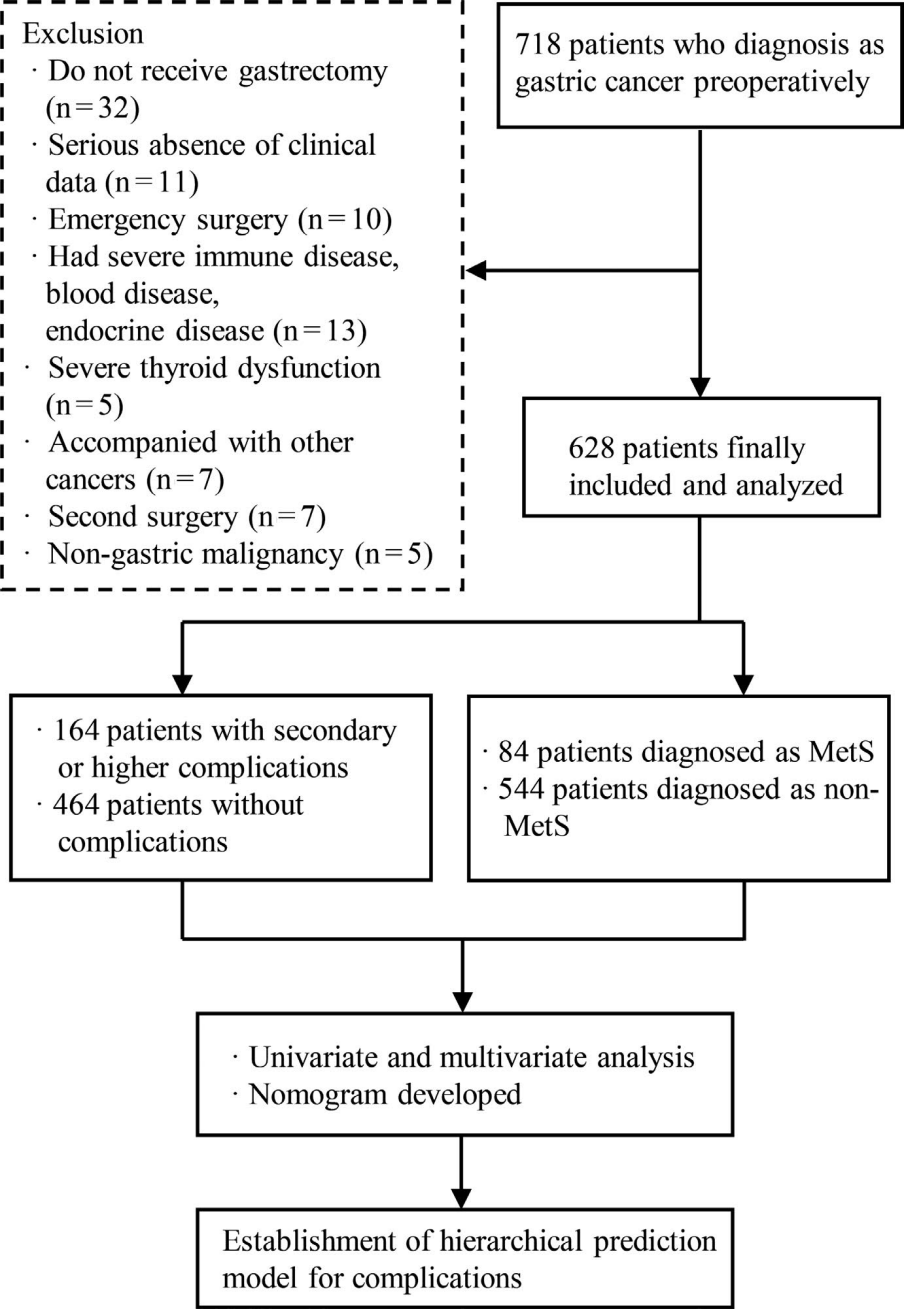
Flow chart of study procedure

GC patients who received radical gastrectomy with D2 lymphadenectomy at the Department of Gastrointestinal Surgery of the Second Affiliated Hospital of Wenzhou Medical University and Yuying children's Hospital between 01 June 2014 and 31 December 2016 were screened for inclusion in the study. The inclusion criteria were as followed: (a) postoperative pathology confirmed gastric adenocarcinoma; (b) aged ≥ eighteen years old; (c) Sign the participation informed consent. The following exclusion criteria were applied: (a) Underwent palliative surgery or emergency surgery; (b) received neoadjuvant chemotherapy or radiotherapy; (c) had a severe immune disease, blood disease, endocrine disease, or thyroid dysfunction; (d) had a history of other malignant tumors; (e) had a history of previous surgery; (f) there was a substantial absence of clinical data. Further details and a flow chart depicting the study procedure are shown in Figure 1.

### 2.2 Baseline data collection

Demographic details including age and sex were collated for each patient, as were details pertaining to the operation such as tumor size, tumor location, and tumor stage. Severe postoperative complications were classified as Clavien‐Dindo^16^ grade II or above in this study.

### 2.3 Diagnosis of MetS

MetS is defined in accordance with the recommendations of the Chinese Diabetes Society^17^ preoperatively, with three or more of the following five items: (a) Body mass index (BMI) determined obesity (BMI≥ 25 kg/cm^2^); (b) with fasting blood glucose above 6.1 mmol/L or diabetes history; (c) systolic blood pressure above 140 mm Hg or diastolic blood pressure ≥90 mm Hg or hypertension history; (d) triglycerides above 1.70 mmol/L; (e) low high‐density lipoprotein cholesterol (<0.9 mmol/L for men, <1.0 mmol/L for women).

### 2.4 Statistical analysis

Parameters Distribution equality was first assessed by Kolmogorov–Smirnov test, means/standard deviation (SD) and medians/interquartile ranges (IQR) were chosed to present normally and non‐normally distributed data, respectively. Chi‐squared test or Fisher's exact test were further performed to compare categorical data. Univariate logistic regression analysis was used and all the variables yielding *p* values < .05 were then selected into multivariable logistic regression (forward stepwise variable selection) to establish the regression model. A nomogram that could quantitatively predict the incidence of secondary or higher complications was constructed, subsequently. Decision curve analysis (DCA) and Receiver operating characteristic (ROC) curve analysis were finally performed to evaluated the clinical usefulness of the nomogram as well as rating model. Statistically significant was determined as *P* < .05. All the statistical analyses were performed using SPSS version 23.0 and R version 4.0.0.

## 3 RESULTS

### 3.1 Baseline characteristics

Among the 718 patients initially included, 90 were subsequently excluded, the reasons for exclusion are detailed in Figure 1. Finally, 628 patients were included in the analysis. Table 1 exhibited the overview of patient population. The mean age of the patients was 62.92 ± 11.45 years, and they included 450 men (71.7%) and 178 women (28.3%).

**Table 1:** Clinical and Pathological Characteristics

Characteristic	Value (N = 628)
Age (y), mean (SD)	62.92 ± 11.45
BMI (kg/cm2), mean (SD)	21.45 ± 3.01
Metabolic disorders [n, (%)]	
No	544 (86.6%)
Yes	84 (13.4%)
Gender [n, (%)]	
Male	450 (71.7%)
Female	178 (28.3%)
Charlson score [n, (%)]	
0	385 (61.3%)
1‐2	220 (35.0%)
3‐6	23 (3.7%)
Preoperative anemia [n, (%)]	
No	517 (82.3%)
Yes	111 (17.7%)
Preoperative hypoalbuminemia [n, (%)]	
No	522 (%)
Yes	106 (%)
Tumor size [n, (%)]	
＜2.1 cm	189 (30.1%)
≥2.1 cm	439 (69.9%)
Tumor location [n, (%)]	
Antrum	550 (87.6%)
Body	50 (8.0%)
Cardia	28 (4.4%)
Histopathological differentiation [n, (%)]	
Differentiation	549 (87.4%)
Non‐ differentiation	49 (7.8%)
Signet ring cell	30 (4.8%)
Lymphatic invasion [n, (%)]	
N0	258 (41.1%)
N1	108 (17.1%)
N2	131 (20.9%)
N3	131(20.9%)
Invasion depth [n, (%)]	
T1/T2	229 (36.5%)
T3/T4	399 (63.5%)
TNM stage [n, (%)]	
I‐II	287 (45.7%)
III‐IV	341 (54.3%)
Abdominal surgery history [n, (%)]	
No	569 (90.6%)
Yes	59(9.4%)
Preoperative obstruction [n, (%)]	
No	536 (85.4%)
Yes	92(14.6%)
Preoperative perforation [n, (%)]	
No	626 (99.7%)
Yes	2 (0.3%)
Preoperative bleeding [n, (%)]	
No	503 (80.1%)
Yes	125 (19.9%)
Anastomosis type [n, (%)]	
Bill‐roth I	236 (37.6%)
Bill‐roth II	349 (55.6%)
Other	43 (6.8%)
Laparoscope [n, (%)]	
No	603 (96.0%)
Yes	25 (4.0%)
Preoperative stroke history [n, (%)]	
No	623 (99.2%)
Yes	5 (0.8%)
CEA [n, (%)]	
<5.0ng/ml	505 (80.4%)
≥5.0ng/ml	123 (19.6%)
CA199 [n, (%)]	
＜37 kU/L	545 (86.8%)
≥37 kU/L	83 (13.2%)

Abbreviation: BMI, bodymass index.

**Figure 2:**
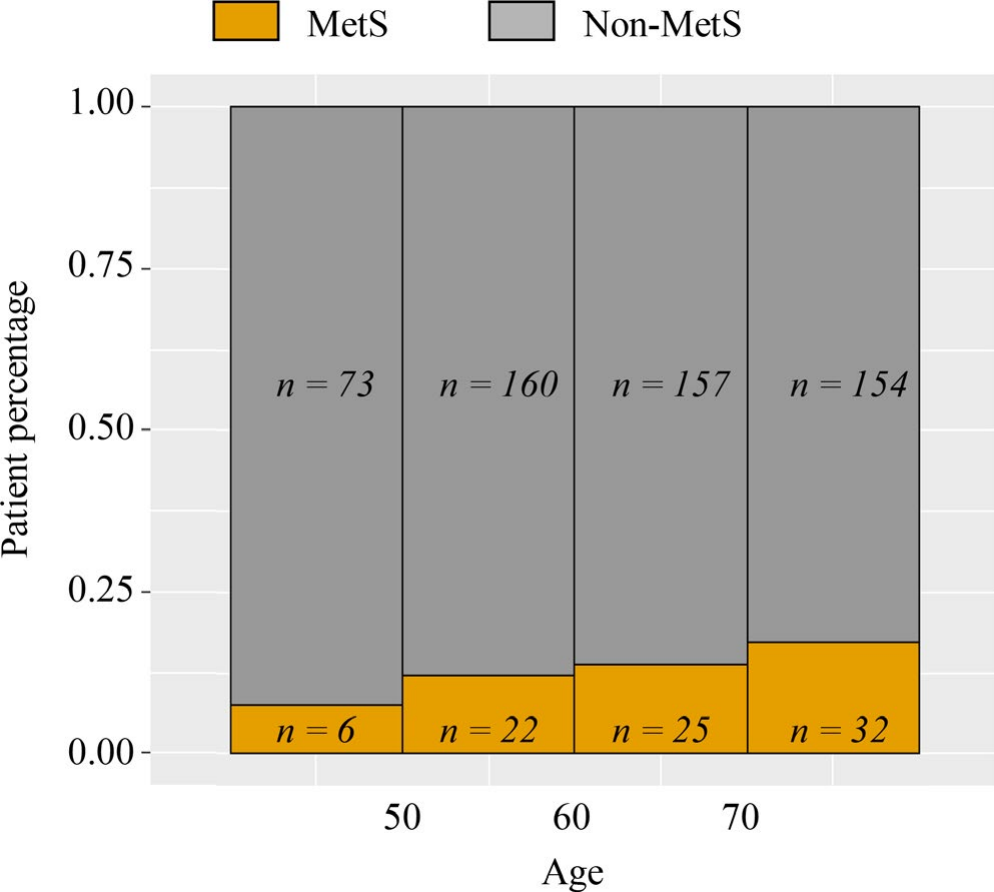
MetS and non‐MetS Patients frequency distribution with different ages stratum

With regard to tumor clinicopathological characteristics, the tumors were located in the antrum in 550/628 patients (87.6%), and 439/628 patients (69.9%) had a tumor that was >2.1 cm along its longest axis. Differentiated tumor histopathology was detected in 549 of the patients (87.4%). Based on the above‐described Chinese Diabetes Society^17^ criteria 84 patients (13.4%) were diagnosed with MetS. The incidence of MetS increased with age, as shown in Figure 2.

### 3.2 Short‐term surgical outcomes

**Figure 3:**
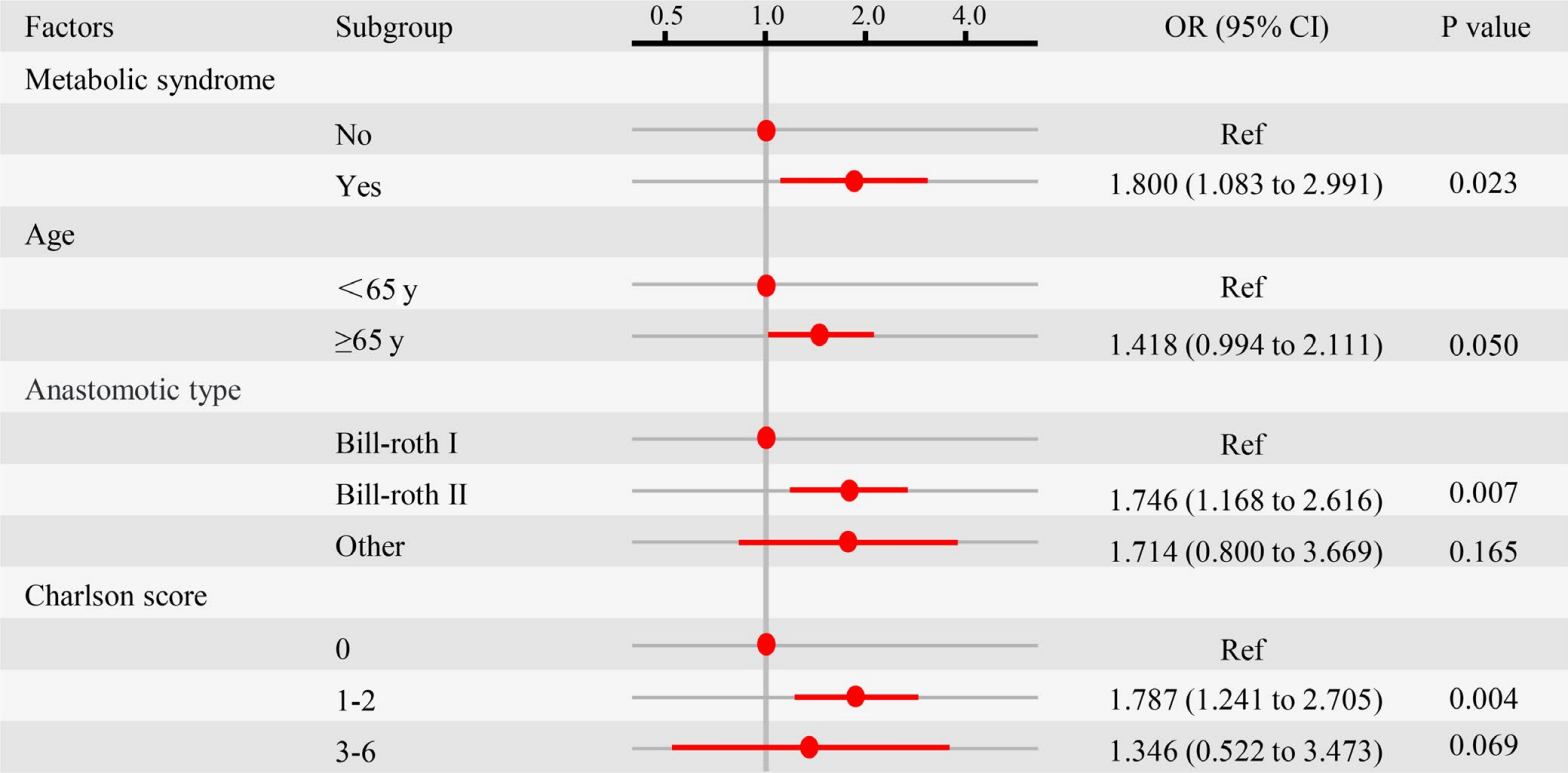
Multivariate analysis to evaluate potential predictive factors for severe postoperative complications

A total of 89 patients (23.4%) experienced severe postoperative complications (Clavien‐Dindo grade ≥ II) (Table 2). The incidence of severe postoperative complications was significantly higher in the MetS group (41.7% versus 23.7%, *P* < .001). In univariate analysis, severe postoperative complications were significantly related to age ≥ 65 years (*P* = .005), metabolic syndrome (*P* = .001), higher Charlson score (*P* < .001 for 1‐2 points), tumor size ≥2.1 cm along the longest axis (*P* = .041), and Billroth II reconstruction (*P* = .003) (Table S1). Severe postoperative complications were not significantly associated with any of the other variables investigated. In multivariate logistic regression analysis MetS (OR 1.800, 95% CI 1.083‐2.991, *P* = .023), age (OR 1.418, 95% CI 0.994‐2.111, *P* = .050), Charlson score (OR 1.787, 95% CI 1.241‐2.705, *P* = .004 for 1‐2 points), and anastomosis type (OR 1.746, 95% CI 1.168‐2.616, *P* = .007 for Billroth II reconstruction) were independent risk factors (Table S1; Figure 3).

**Table 2:** Postoperative outcomes

Factors	Total (n = 628)	MetS (n = 84)	Non‐MetS (n = 544)	*P*
Total complications^a^	164 (26.11%)	35 (41.67%)	129 (23.71%)	＜.001*
Detail of complications				
Surgical complications				
Gastroparesis	23 (3.66%)	9 (10.71%)	14 (2.57%)	＜.001*
Intestinal obstruction	14 (2.23%)	1 (1.19%)	13 (2.39%)	.453
Severe wound infection	10 (1.59%)	2 (2.38%)	8 (1.47%)	.558
Bleeding	14 (2.23%)	3 (3.57%)	11 (2.02%)	.403
Intra‐abdominal infection	22 (3.50%)	4 (4.76%)	18 (3.31%)	.500
Anastomotic leakage	9 (1.43%)	1 (1.19%)	8 (1.47%)	.841
Medical complications				
Pulmonary complications	16 (2.55%)	4 (4.76%)	12 (2.21%)	.207
Pleural and peritoneal effusion	11 (1.75%)	5 (5.95%)	6 (1.10%)	.002*
Cardiac complications	3 (0.48%)	0 (0.00%)	3 (0.55%)	.353
Venous thrombosis	12 (1.91%)	2 (2.38%)	10 (1.84%)	.743
Second operation	1 (0.16%)	0 (0.00%)	1 (0.18%)	.592
Complex complications (two or more complications)	29 (4.62%)	4 (4.76%)	25 (4.60%)	.946

^a^ Clavien‐Dindo grade ≥ II.

* Statistically significant (*P* < .05).

Of the 628 gastric cancer patients, 331 (52.7%) were aged <65 years (“younger” group) and the remaining 297 (47.3%) were aged ≥65 years (“older” group). Of the 331 patients in the younger group, 35 (10.6%) were diagnosed with MetS and 71 (21.5%) developed severe postoperative complications. Of the 297 patients in the older group, 49 (16.5%) were diagnosed with MetS and 93 (31.3%) developed severe postoperative complications. Interestingly, MetS was an independent risk factor for severe postoperative complications in the younger group (OR 2.235, 95% CI 1.015‐4.921, *P* = .046) but not in the older group (Table S2).

### 3.3 Nomogram development and usefulness

**Figure 4:**
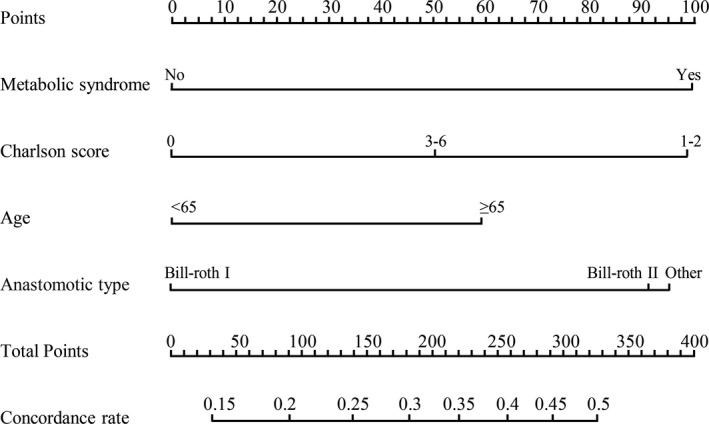
Developed nomogram for predicting postoperative complications in GC patients

**Figure 5:**
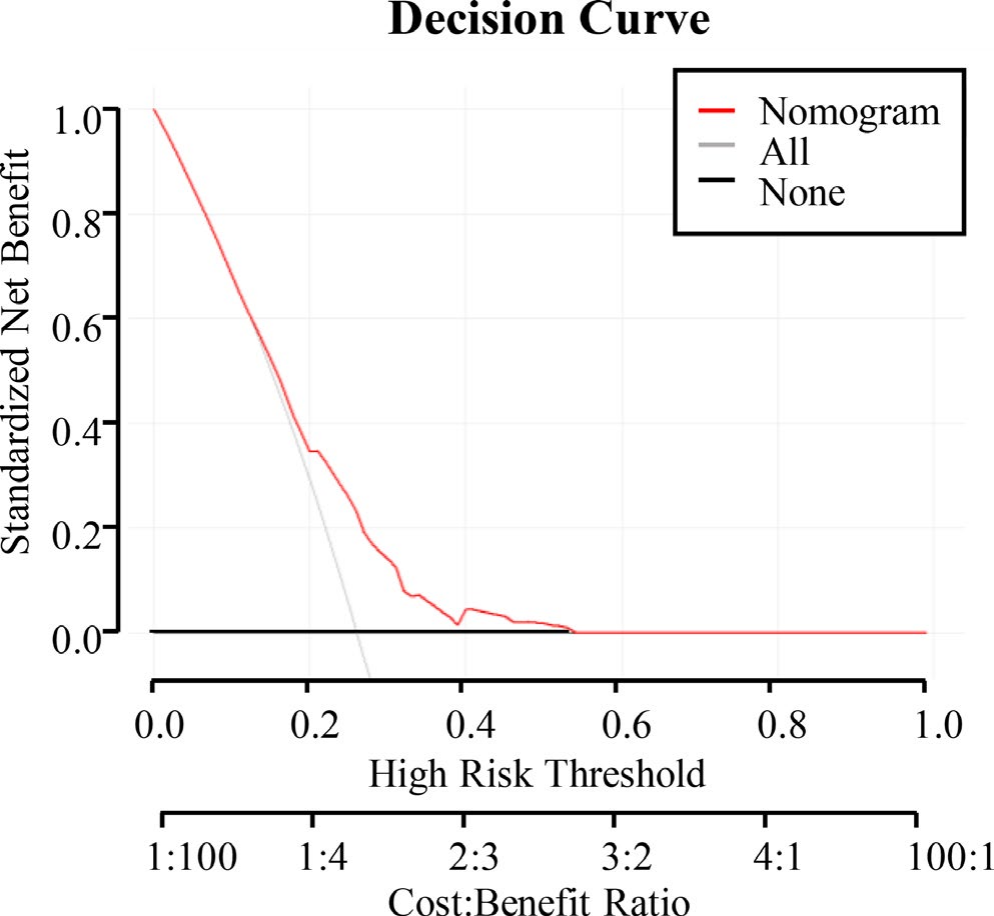
DCA for the nomogram. Decision curve analysis for the nomogram. The y‐axis measures the net benefit. The red line represents the nomogram. The gray line represents the assumption that all cases were concordant and The black line represents the assumption that no cases were concordant

A model incorporating the independent predictors that yielded *P* < .05 in multivariate analysis was developed and structured as a nomogram (Figure 4). Further DCA demonstrated that a use the nomogram adds more benefit than either the treat all or treat none scheme while the threshold probability of a patient is 13% to 54% (Figure 5). For example, if the personal threshold probability of a patient is 30% (*ie*, the patient would opt for treatment if their probability of postoperative complications was 30%), then the net benefit when using the nomogram to make a decision as to whether to undergo treatment is 0.246, constituting added benefit compared with the treat‐all or treat‐none scheme.

### 3.4 Development of postoperative complications risk‐rating model

**Figure 6:**
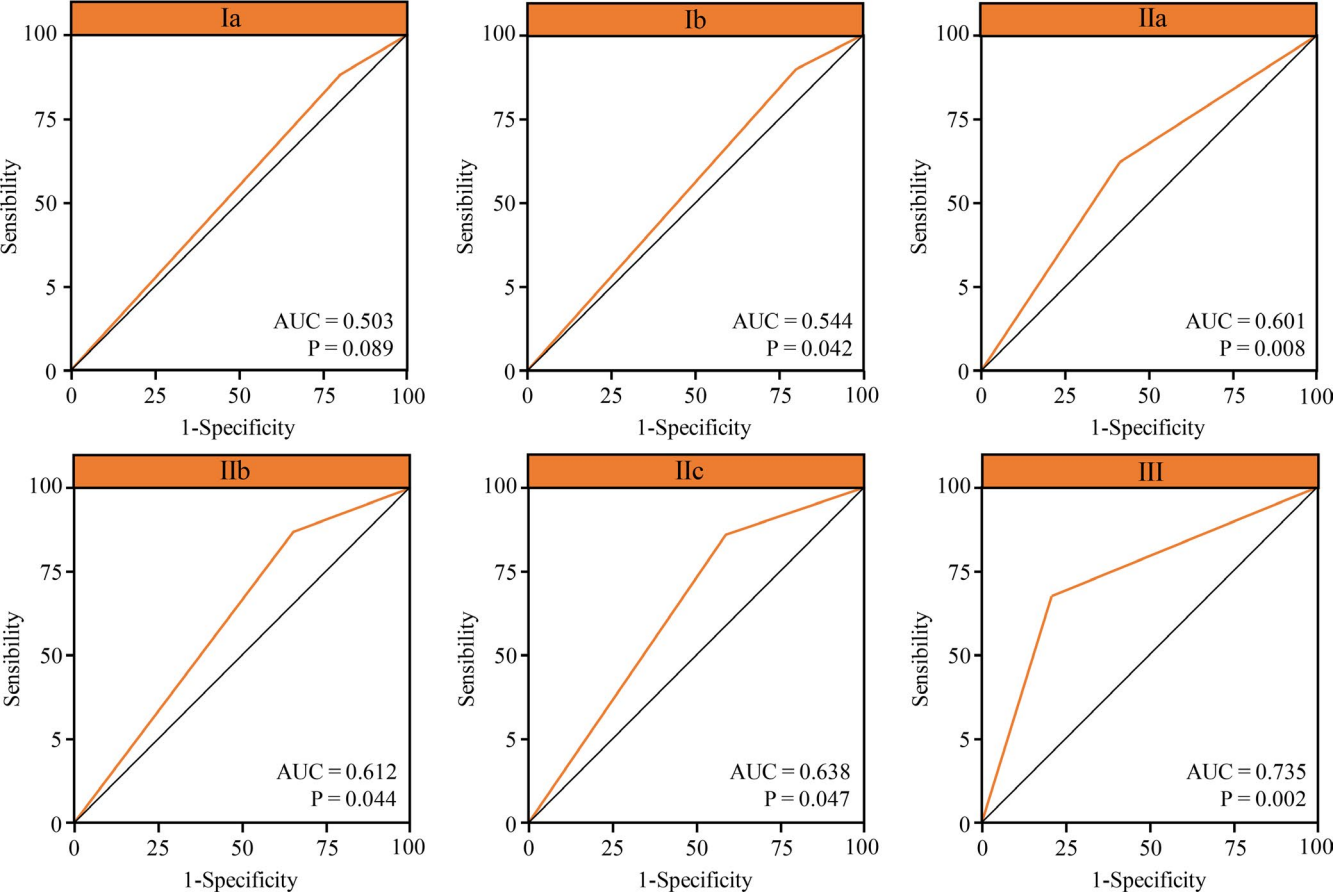
ROC curves for the postoperative complications risk‐rating model

Based on the univariate analyses, a postoperative complications risk‐rating model was further developed (Table 3). ROC analyses showed that higher risk‐rating predicted the occurrence of complications more accurately with a higher area under the curve (AUC) (ROC I = 0.503, ROC Ib = 0.544, ROC IIa = 0.601, ROC IIb = 0.612, ROC IIc = 0.638, ROC III = 0.735) (Figure 6).

**Table 3:** The risk rating models for postoperative complications

level	Conditions
0	Tumor size < 2.1cm Age < 65 years No preoperative metabolic syndrome Preoperative Charlson score = 0 points Not the Billroth II reconstruction
Ⅰa	Tumor size > 2.1cm Age > 65 years No preoperative metabolic syndrome Preoperative Charlson score = 0 points Not the Billroth II reconstruction
Ⅰb	Regardless of tumor size, meet one of the four conditions as Age > 65 years preoperative metabolic syndrome Preoperative Charlson score > 0 points Billroth II reconstruction
Ⅱa	Tumor size < 2.1cm meet two of the four conditions as Age > 65 years preoperative metabolic syndrome Preoperative Charlson score > 0 points Billroth II reconstruction
Ⅱb	Tumor size > 2.1cm meet two of the four conditions as Age > 65 years preoperative metabolic syndrome Preoperative Charlson score > 0 points Billroth II reconstruction
Ⅱc	Tumor size > 2.1cm meet three of the four conditions as Age > 65 years preoperative metabolic syndrome Preoperative Charlson score > 0 points Billroth II reconstruction
Ⅲ	Tumor size > 2.1cm Age > 65 years preoperative metabolic syndrome Preoperative Charlson score > 0 points Billroth II reconstruction

## 4 DISCUSSION

Considering the growing incidence rate of GC and the ongoing obesity epidemic in China, researchers have investigated associations between MetS and GC. Lin et al^18^ demonstrated that MetS is relevant to GC in both women and men, and Kim ^19^ reported that patients with more than three risk factors for MetS were more probably to suffer GC. Dan Hu et al^2^ reported that preoperative MetS was predictive factor for mortality after radical gastrectomy, particularly those with early GC. To our knowledge however, the current investigation is the first cohort study to investigate associations between preoperative MetS and postoperative complications after gastrectomy.

In this study, the MetS incidence as diagnosed in accordance with the Chinese Diabetes Society criteria^17^ was 13.4%, which is comparable to that previously reported.^7^ MetS was further turned out to be an independent risk factor for severe postoperative complications after gastrectomy as expected. Given that MetS is consist of a bunch of metabolic abnormalities such as obesity, dyslipidemia, hypertension, and hyperglycemia, insulin resistance may be the most probable pathophysiological cause.

Mounting evidence suggests that insulin resistance influences abnormal metabolism in adipocytes, with succeeding increasing of pro‐inflammatory cytokines and decreaing of adiponectin (protective adipokine),^20^ thus leading to infective complications including infection of surgical site, abdominal infection, and pneumonia. Abdominal obesity is also reportedly correlated with insulin resistance—and thus reduced oxygen tension within surgical wounds—which can give rise to infectious complications.^21^ Furthermore, the massive and fragile fat tissue in abdominally obese patients is easily torn and greatly reduces surgical precision,^22^ which could prolong the surgery duration and increase intraoperative hemorrhage, resulting in an increased risk of wound infection. In another study hypertension and dyslipidemia were both related to poor microvascular circulation,^23^ which impaired tissue repair and increased the risk of wound complications such as anastomotic leakage. Although each individual component may only have a small influence, the risk of postoperative complications was substantially increased when these minor forms of damage occurred together.

As has been reported previously,^2^, ^24^ we also found the incidence rate of MetS was higher in the elderly. Age was another independent risk factor for postoperative complications in the present study. Considering the difference in physical condition between younger patients and older patients, we investigated whether MetS correlated with outcome separately in the younger and older groups. Interestingly MetS could only independently predicted postoperative complications in the young. It may be that the value of MetS predicting postoperative complications in the elderly is reduced by age and age‐related comparatively poorer physical condition. Thus under the same diagnostic criteria, more attention should be paid to young patients.

In the present study, Billroth II anastomosis was also an independent risk factor for postoperative complications in our study, consistent with Kang KC demonstrated that the postoperative complication was much less in a Billroth I group than in a Billroth II group.^25^ Notably however, in another study there was no statistical differences in morbidity derived from postoperative complications in a Billroth I group and a Billroth II group. Despite of differences in operation technique, although Billroth II reconstruction was tension‐free with the application of anastomosis of gastric‐jejunum, it has the disadvantages of altering the digestive tract and taking longer time to empty, which potentially leading to duodenal stump leakage.^26^, ^27^ Consistent with previous reports,^28^, ^29^ Charlson score was independently relevant to postoperative complications in the current study.

The nomogram incorporating MetS, anastomotic type, age, and Charlson score to predict individual risk of postoperative complications developed in the current study has potential clinical applications. Patients at a high risk of developing postoperative complications could be monitored more closely. With this aim, DCA, which offers insight into clinical consequences based on net benefit derived threshold probability, was applied in this study. The net benefit was considered as the difference between proportion of true positives and false positives and weighted by the relative harm of false positive and false negative. DCA demonstrated that while a patient's threshold probability determined by the nomogram developed in the present study is > 13% and < 54%, offering prophylactic measures adds more benefit than either the treat all or treat none scheme.

A postoperative complications risk rating model was finally developed in the present study. Further ROC analysis showed that a higher risk rating corresponded with a higher AUC indicating that the model could be used effectively to screen susceptible patients of postoperative complications. The current study is the first to investigate relationships between MetS and postoperative complications, and also first constructed the nomogram to quantify the possibility of postoperative complications.

The present study had several limitations. Although it can be considered representative because the patients in the study were prospectively enrolled in two large centers, the conclusions of the study is needed to confirm via a randomized controlled multicenter study. Another limitation is that the study only included short term outcomes (within 30 days postoperatively). Long term follow‐up data are also needed.

In conclusion, it is the first report of relationship between preoperative MetS and short‐term prognosis. MetS, as well as Billroth II anastomosis, age, and Charlson score affected postoperative complications after gastrectomy. A nomogram incorporating these independent risk factors was developed. Preoperative use of this prediction tool could provide useful information to surgeons with respect to better preoperative preparation to decrease the risks associated with the surgery economically and conveniently.

## ETHICS APPROVAL AND CONSENT TO PARTICIPATE

All participants provided their written informed consent, and the protocol for this study was approved by the ethics committee of The Second Affiliated Hospital of Wenzhou Medical University and Yuying children's Hospital.

## CONFLICT OF INTERESTS

The authors declare that they have no competing interests, and all authors should confirm its accuracy.

## AUTHOR CONTRIBUTIONS

Study concepts: XDC, Study design: WTZ and XWS, Data acquisition: CCM and MMS, Data analysis and interpretation: LBX, YQC, Statistical analysis: WJC and XDC, Manuscript preparation: XDC and WTZ, Manuscript editing: CCM, Manuscript review: XS.

## Supporting information

Table S1

Table S2

## Data Availability

The datasets used during the current study are available from the corresponding author on reasonable request.
