# Supplementary material for: Metabolic syndrome predicts postoperative complications after gastrectomy in gastric cancer patients: Development of an individualized usable nomogram and rating model
Source: Cancer Med. 2020 Aug 17;9(19):7116–24. doi: 10.1002/cam4.3352 (PMC7541147; doi:10.1002/cam4.3352)
Supplement: Supplementary file 1 — Table S1 [file CAM4-9-7116-s001.docx]

Table. S1 Univariate and multivariate analysis of risk factors for postoperative complications

| Characteristic | Univariate analysis | | Multivariate analysis | |
| --- | --- | --- | --- | --- |
|  | OR (95% CI) | P | OR (95% CI) | P |
| Age |  |  |  |  |
| ＜65 | 1 |  | 1 |  |
| ≥65 | 1.669(1.166-2.391) | 0.005* | 1.418(0.994-2.111) | 0.050* |
| Gender |  |  |  |  |
| Female | 1 |  |  |  |
| Male | 1.155(0.773-1.725) | 0.483 |  |  |
| Metabolic disorders |  |  |  |  |
| No | 1 |  | 1 |  |
| Yes | 2.298(1.427-3.701) | 0.001* | 1.800(1.083-2.991) | 0.023* |
| Charlson score |  |  |  |  |
| 0 | 1 |  | 1 |  |
| 1-2 | 2.128(1.468-3.083) | ＜0.001* | 1.787(1.241-2.705) | 0.004* |
| 3-6 | 1.695(0.674-4.261) | 0.262 | 1.346(0.522-3.473) | 0.069 |
| Preoperative anemia |  |  |  |  |
| No | 1 |  |  |  |
| Yes | 0.892(0.554-1.434) | 0.636 |  |  |
| Preoperative hypoalbuminemia |  |  |  |  |
| No | 1 |  |  |  |
| Yes | 1.278(0.807-2.022) | 0.296 |  |  |
| Tumor location |  |  |  |  |
| Antrum | 1 |  |  |  |
| Body | 1.365(0.731-2.548) | 0.329 |  |  |
| Cardia | 0.967(0.402-2.323) | 0.940 |  |  |
| Histopathological differentiation |  |  |  |  |
| Differentiation | 1 |  |  |  |
| Non- differentiation | 1.125(0.588-2.151) | 0.722 |  |  |
| Signet ring cell | 0.703(0.282-1.755) | 0.450 |  |  |
| Tumor size |  |  |  |  |
| ＜2.1 cm | 1 |  | 1 |  |
| ≥2.1 cm | 1.531(1.017-2.304) | 0.041* | 1.361(0.886-2.091) | 0.159 |
| Lymphatic invasion |  |  |  |  |
| N0 | 1 |  |  |  |
| N1 | 1.615(0.987-2.642) | 0.057 |  |  |
| N2 | 1.132(0.697-1.838) | 0.616 |  |  |
| N3 | 1.087(0.668-1.771) | 0.736 |  |  |
| Invasion depth |  |  |  |  |
| T1/T2 | 1 |  |  |  |
| T3/T4 | 1.328(0.910-1.939) | 0.142 |  |  |
| TNM stage |  |  |  |  |
| I-II | 1 |  |  |  |
| III-IV | 1.220(0.852-1.748) | 0.278 |  |  |
| Abdominal surgery history |  |  |  |  |
| No | 1 |  |  |  |
| Yes | 0.782(0.411-1.488) | 0.454 |  |  |
| Preoperative obstruction |  |  |  |  |
| No | 1 |  |  |  |
| Yes | 1.540(0.958-2.475) | 0.075 |  |  |
| Preoperative bleeding |  |  |  |  |
| No | 1 |  |  |  |
| Yes | 0.779(0.490-1.239) | 0.292 |  |  |
| Anastomosis type |  |  |  |  |
| Bill-roth I | 1 |  | 1 |  |
| Bill-roth II | 1.802(1.214-2.674) | 0.003* | 1.746(1.168-2.616) | 0.007* |
| Other | 1.599(0.763-3.352) | 0.214 | 1.714(0.800-3.669) | 0.165 |
| Laparoscope |  |  |  |  |
| No | 1 |  |  |  |
| Yes | 0.514(0.855-4.417) | 0.113 |  |  |
| Preoperative stroke history |  |  |  |  |
| No | 1 |  |  |  |
| Yes | 1.897(0.314-11.455) | 0.485 |  |  |
| CEA |  |  |  |  |
| <5.0ng/ml | 1 |  |  |  |
| ≥5.0ng/ml | 0.893(0.566-1.410) | 0.627 |  |  |
| CA199 |  |  |  |  |
| ＜ 37 kU/L | 1 |  |  |  |
| ≥ 37 kU/L | 1.436(0.872-2.363) | 0.155 |  |  |

OR, odds ratio; CI, confidence interval

*Statistically significant (P<0.05)
