# Supplementary material for: Metabolic syndrome predicts postoperative complications after gastrectomy in gastric cancer patients: Development of an individualized usable nomogram and rating model
Source: Cancer Med. 2020 Aug 17;9(19):7116–24. doi: 10.1002/cam4.3352 (PMC7541147; doi:10.1002/cam4.3352)
Supplement: Supplementary file 2 — Table S2 [file CAM4-9-7116-s002.docx]

Table. S2 Univariate and multivariate analysis of risk factors for postoperative complications in young patients and elderly patients

| Characteristic | Young patients (age <65 ) (n=331) | | | | Old patients (age ≥ 65) (n=297) | | | |
| --- | --- | --- | --- | --- | --- | --- | --- | --- |
|  | Univariate analysis | | Multivariate analysis | | Univariate analysis | | Multivariate analysis | |
|  | OR (95% CI) | P | OR (95% CI) | P | OR (95% CI) | P | OR (95% CI) | P |
| Gender |  |  |  |  |  |  |  |  |
| Female | 1 |  |  |  | 1 |  |  |  |
| Male | 0.955(0.541-1.686) | 0.873 |  |  | 1.329(0.749-2.361) | 0.331 |  |  |
| Metabolic disorders |  |  |  |  |  |  |  |  |
| No | 1 |  | 1 |  | 1 |  |  |  |
| Yes | 3.214(1.549-6.669) | 0.002* | 2.235(1.015-4.921) | 0.046 | 1.653(0.879-3.110) | 0.119 |  |  |
| Charlson score |  |  |  |  |  |  |  |  |
| 0 | 1 |  |  |  | 1 |  | 1 |  |
| 1-2 | 2.327(1.330-4.070) | 0.003* | 1.979(1.091-3.591) | 0.025* | 1.715(1.033-2.847) | 0.037* | 1.747(1.048-2.913) | 0.033* |
| 3-6 | 3.731(0.803-17.33) | 0.093 | 2.707(0.534-13.73) | 0.229 | 0.933(0.285-3.016) | 0.909 | 0.998(0.303-3.283) | 0.997 |
| Preoperative anemia |  |  |  |  |  |  |  |  |
| No | 1 |  |  |  | 1 |  |  |  |
| Yes | 0.703(0.298-1.658) | 0.421 |  |  | 0.867(0.480-1.563) | 0.634 |  |  |
| Preoperative hypoalbuminemia |  |  |  |  |  |  |  |  |
| No | 1 |  |  |  | 1 |  |  |  |
| Yes | 0.628(0.233-1.689) | 0.357 |  |  | 1.405(0.806-2.448) | 0.230 |  |  |
| Tumor location |  |  |  |  |  |  |  |  |
| Antrum | 1 |  |  |  | 1 |  |  |  |
| Body | 1.420(0.568-3.550) | 0.454 |  |  | 1.291(0.547-3.047) | 0.560 |  |  |
| Cardia | 0.304(0.039-2.384) | 0.257 |  |  | 1.530(0.527-4.445) | 0.435 |  |  |
| Histopathological differentiation |  |  |  |  |  |  |  |  |
| Differentiation | 1 |  |  |  | 1 |  |  |  |
| Non- differentiation | 0.967(0.310-3.014) | 0.953 |  |  | 1.081(0.484-2.416) | 0.849 |  |  |
| Signet ring cell | 0.837(0.231-3.025) | 0.786 |  |  | 0.590(0.160-2.172) | 0.427 |  |  |
| Tumor size |  |  |  |  |  |  |  |  |
| ＜2.1 cm | 1 |  |  |  | 1 |  |  |  |
| ≥2.1 cm | 1.348(0.763-2.382) | 0.303 |  |  | 1.577(0.866-2.871) | 0.137 |  |  |
| Lymphatic invasion |  |  |  |  |  |  |  |  |
| N0 | 1 |  |  |  | 1 |  |  |  |
| N1 | 1.162(0.547-2.469) | 0.696 |  |  | 1.923(0.972-3.805) | 0.060 |  |  |
| N2 | 0.710(0.336-1.502) | 0.370 |  |  | 1.543(0.786-3.030) | 0.208 |  |  |
| N3 | 0.944(0.460-1.937) | 0.875 |  |  | 1.154(0.582-2.287) | 0.682 |  |  |
| Invasion depth |  |  |  |  |  |  |  |  |
| T1/T2 | 1 |  |  |  | 1 |  |  |  |
| T3/T4 | 0.997(0.588-1.690) | 0.992 |  |  | 1.521(0.852-2.714) | 0.156 |  |  |
| TNM stage |  |  |  |  |  |  |  |  |
| I-II | 1 |  |  |  | 1 |  |  |  |
| III-IV | 0.746(0.438-1.270) | 0.280 |  |  | 1.677(0.989-2.842) | 0.055 |  |  |
| Abdominal surgery history |  |  |  |  |  |  |  |  |
| No | 1 |  |  |  | 1 |  |  |  |
| Yes | 0.909(0.329-2.514) | 0.854 |  |  | 0.644(0.280-1.483) | 0.301 |  |  |
| Preoperative obstruction |  |  |  |  |  |  |  |  |
| No | 1 |  |  |  | 1 |  |  |  |
| Yes | 1.306(0.603-2.826) | 0.498 |  |  | 1.568(0.847-2.901) | 0.152 |  |  |
| Preoperative bleeding |  |  |  |  |  |  |  |  |
| No | 1 |  |  |  | 1 |  |  |  |
| Yes | 0.699(0.344-1.421) | 0.323 |  |  | 0.838(0.449-1.564) | 0.578 |  |  |
| Anastomosis type |  |  |  |  |  |  |  |  |
| Bill-roth I | 1 |  | 1 |  | 1 |  |  |  |
| Bill-roth II | 2.066(1.170-3.650) | 0.012* | 2.051(1.146-3.670) | 0.016* | 1.506(0.865-2.622) | 0.147 |  |  |
| Other | 0.425(0.053-3.423) | 0.421 | 0.503(0.062-4.107) | 0.522 | 1.809(0.753-4.346) | 0.185 |  |  |
| Laparoscope |  |  |  |  |  |  |  |  |
| No | 1 |  |  |  | 1 |  | 1 |  |
| Yes | 1.103(0.295-4.120) | 0.884 |  |  | 0.309(0.095-1.000) | 0.050* | 3.345(1.019-10.98) | 0.047* |
| CEA |  |  |  |  |  |  |  |  |
| <5.0ng/ml | 1 |  |  |  | 1 |  |  |  |
| ≥5.0ng/ml | 0.789(0.387-1.612) | 0.516 |  |  | 0.934(0.510-1.709) | 0.824 |  |  |
| CA199 |  |  |  |  |  |  |  |  |
| ＜ 37 kU/L | 1 |  |  |  | 1 |  |  |  |
| ≥ 37 kU/L | 1.470(0.589-3.673) | 0.409 |  |  | 1.197(0.652-2.197) | 0.562 |  |  |

OR, odds ratio; CI, confidence interval

*Statistically significant (P<0.05)
